# Improvement of clinical wound microcirculation diagnosis using an object tracking-based laser speckle contrast imaging system

**DOI:** 10.1063/5.0172443

**Published:** 2024-01-29

**Authors:** Meng-Che Hsieh, Chia-Yu Chang, Ching-Han Hsu, Yan-Ren Lin, Pei-You Hsieh, Congo Tak-Shing Ching, Lun-De Liao

**Affiliations:** 1 Institute of Biomedical Engineering and Nanomedicine, National Health Research Institutes, 35, Keyan Road, Zhunan Town, Miaoli County 35053, Taiwan; 2 Program in Tissue Engineering and Regenerative Medicine, National Chung Hsing University, 145, Xingda Road, South District, Taichung 402, Taiwan; 3 Department of Biomedical Engineering and Environmental Sciences, National Tsing Hua University, No. 101, Section 2, Kuang-Fu Road, Hsinchu 30013, Taiwan; 4 Department of Emergency and Critical Care Medicine, Changhua Christian Hospital, Changhua, Taiwan; 5 Graduate Institute of Biomedical Engineering, National Chung Hsing University, 145, Xingda Road, South District, Taichung 402, Taiwan; 6 Department of Electrical Engineering, National Chi Nan University, 1 University Road, Puli Township, Nantou County 545301, Taiwan

## Abstract

Wound monitoring is crucial for effective healing, as nonhealing wounds can lead to tissue ulceration and necrosis. Evaluating wound recovery involves observing changes in angiogenesis. Laser speckle contrast imaging (LSCI) is vital for wound assessment due to its rapid imaging, high resolution, wide coverage, and noncontact properties. When using LSCI equipment, regions of interest (ROIs) must be delineated in lesion areas in images for quantitative analysis. However, patients with serious wounds cannot maintain constant postures because the affected areas are often associated with discomfort and pain. This leads to deviations between the drawn ROI and actual wound position when using LSCI for wound assessment, affecting the reliability of relevant assessments. To address these issues, we used the channel and spatial reliability tracker object tracking algorithm to develop an automatic ROI tracking function for LSCI systems. This algorithm is used to track and correct artificial movements in blood flow images, address the ROI position offset caused by the movement of the affected body part, increase the blood flow analysis accuracy, and improve the clinical applicability of LSCI systems. ROI tracking experiments were performed by simulating wounds, and the results showed that the intraclass correlation coefficient (ICC) ranged from 0.134 to 0.976. Furthermore, the object within the ROI affected tracking performance. Clinical assessments across wound types showed ICCs ranging from 0.798 to 0.917 for acute wounds and 0.628–0.849 for chronic wounds. We also discuss factors affecting tracking performance and propose strategies to enhance implementation effectiveness.

## I. INTRODUCTION

### A. Research background information

In clinical practice, careful monitoring and assessment of patients' wounds are crucial for the wound healing process. Prolonged nonhealing of a wound can lead to skin ulceration and necrosis. The wound healing process can be divided into three phases, the inflammatory phase, proliferative phase, and remodeling phase, which are not independent and often overlap.^1,2^ During the inflammatory phase, blood vessels rupture, leading to bleeding. The immune system activates defense mechanisms to combat external bacteria and microorganisms, resulting in inflammatory reactions characterized by redness, swelling, heat, and pain at the wound site. The inflammatory phase typically lasts for hours to days. The proliferative phase primarily involves the growth of granulation tissue to fill the wound. During this stage, cells generate collagen to close the wound, and new blood vessels gradually form at the wound site, providing nutrients and oxygen to aid in subsequent wound repair. Providing a clean and moist environment for the wound during this stage can accelerate tissue repair and expedite wound healing. After wound closure, the wound enters the remodeling phase. In this stage, excess microvessels regress, and the color of the granulation tissue gradually transitions to a hue similar to that of the surrounding skin. Irregularly arranged collagen caused by the injury is replaced by newly synthesized and aligned collagen fibers, leading to soft and smooth scar tissue. The remodeling phase can last for months to years, and if healing is disrupted, improper collagen alignment may lead to scar formation. Among these three phases, the proliferative phase is considered the most crucial. The establishment and shaping of new blood vessels are critical for proper wound healing. The nutrients needed for granulation and new tissue generation are supplied by microvessels. Neovascularization failure can impede wound healing, leading to chronic ulcers and, in severe cases, necrosis with blackening. However, these vascular changes cannot be directly observed by eye, necessitating the use of observation instruments.

### B. Microvascular blood flow detection technologies

Currently, the most commonly used instruments for microcirculation observation in clinical practice include microvascular imaging devices^3–7^ and laser Doppler flowmetry.^8–10^ Microvascular imaging devices are primarily used for microscopically visualizing dynamic processes of capillaries in human nail folds, allowing clear observation of the shape and blood flow status of microvessels. However, users need to undergo professional training and have certain experience to accurately differentiate different microvessels. Laser Doppler flowmetry, based on the Doppler principle, analyzes the blood flow status by detecting the Doppler shift caused by moving red blood cells and scattered light. Laser Doppler flowmetry is often used for continuous monitoring of microvascular blood perfusion and blood flow changes. In laser Doppler flowmetry systems, a fiber optic probe needs to be fixed to the tissue surface. However, this type of probe needs to be in contact with the monitoring site, limiting its applicability to open wounds, as the probe can only be attached near the affected area in such cases. Moreover, this type of probe cannot be used to measure actual blood flow changes within a wound. Thus, microvascular circulation assessment methods may not be reliable for patient wounds.

Laser speckle contrast imaging (LSCI) is an emerging optical imaging technique that enables dynamic visualization of blood flow and perfusion in large tissue areas.^11,12^ When coherent light is scattered by a medium, the scattered light undergoes interference and superposition, forming a speckle pattern. Continuous displacement of scatterers within the medium leads to changes in the speckle pattern. By capturing images with a certain exposure time, the pattern exhibits varying degrees of brightness, contrast, and blurriness. Speckle contrast patterns can be quantified to estimate the concentration and velocity of moving particles (primarily red blood cells in the case of tissue imaging), thereby providing a quantitative assessment of red blood cell motion in the skin. Currently, LSCI technology has been widely applied to measure blood microcirculation parameters in organ tissues such as the skin, brain,^13,14^ and retina.^15^ LSCI technology provides clear tissue blood flow imaging data and has advantages such as a short imaging time, high resolution, large imaging area, and noncontact measurement capability. Therefore, LSCI is well suited for wound assessment. LSCI technology is commonly used in the treatment of severe injuries such as burns^16^ and diabetic foot ulcers.^17,18^ LSCI systems assist physicians in quantitatively evaluating blood flow and perfusion in the wound area, assessing the impact of treatment on blood flow and perfusion around the wound, predicting the wound healing speed, and developing more effective treatment plans.

### C. Research motivation

When LSCI technology is used for clinical assessment, the regions of interest (ROIs) within the imaged area must be delineated to quantitatively analyze blood flow patterns. Unfortunately, for patients with wounds such as burns, diabetic foot injuries, and pressure ulcers, the affected areas are often associated with discomfort and pain, making it difficult for patients to maintain a steady posture. As a result, when evaluating wounds using LSCI, the drawn ROI positions may deviate from the actual wound locations, thereby affecting the reliability of the wound blood flow assessment. Additionally, in clinical practice, various tests are typically performed to assess vascular functionality, such as the postocclusive reactive hyperemia (PORH) test^19^ and Buerger's test.^20^ During these tests, the tested area of the patient inevitably moves and is displaced. A common approach for addressing the movement of the affected area is manually repositioning the ROI in each image frame to compensate for the displacement during the measurement. However, this manual process of repeatedly drawing ROIs is time-consuming, significantly limiting the clinical applicability of LSCI systems. Therefore, to address this drawback, an LSCI system that can assist in ROI localization during the measurement process, thereby allowing the ROI position to be tracked even when the wound moves, should be developed.

### D. Research purpose

To address the above-mentioned technical difficulties, we developed an automatic ROI tracking function for LSCI systems. This function is mainly aimed at tracking and correcting artificial movement in blood flow images to address the ROI position offset caused by clinical patient discomfort and movement of affected parts, improve image quality and blood flow analysis accuracy, and enhance the clinical applicability of LSCI systems. In this study, we used the discriminative correlation filter with channel and spatial reliability tracker (CSRT) algorithm for object tracking. Among the various object tracking algorithms included in OpenCV, the CSRT algorithm shows excellent performance in terms of accuracy. To realize multiobject tracking, we designed multiple object trackers in the system, and the number of trackers can be set according to the experimental requirements to realize automatic tracking of multiple ROIs. This function is expected to improve the accuracy and efficiency of blood flow analysis and has potential for use in clinical applications.

## II. RESULTS

### A. Results of the affected part movement simulation experiment

In this experiment, we simulated and moved wounds to study the ROI tracking performance of the CSRT. We obtained three sets of images for each location, namely, the back of the hand and the forearm, with a recording time of 30 s per set. During the automatic tracking experiment, the algorithm analyzed each speckle image frame, and ROIs needed to be delineated in only the first frame, which took an average of approximately 10 s. In contrast, during the manual tracking experiment, the ROIs had to be repositioned in each speckle image frame, which took an average of approximately 10 min per iteration.

**FIG. 1.**
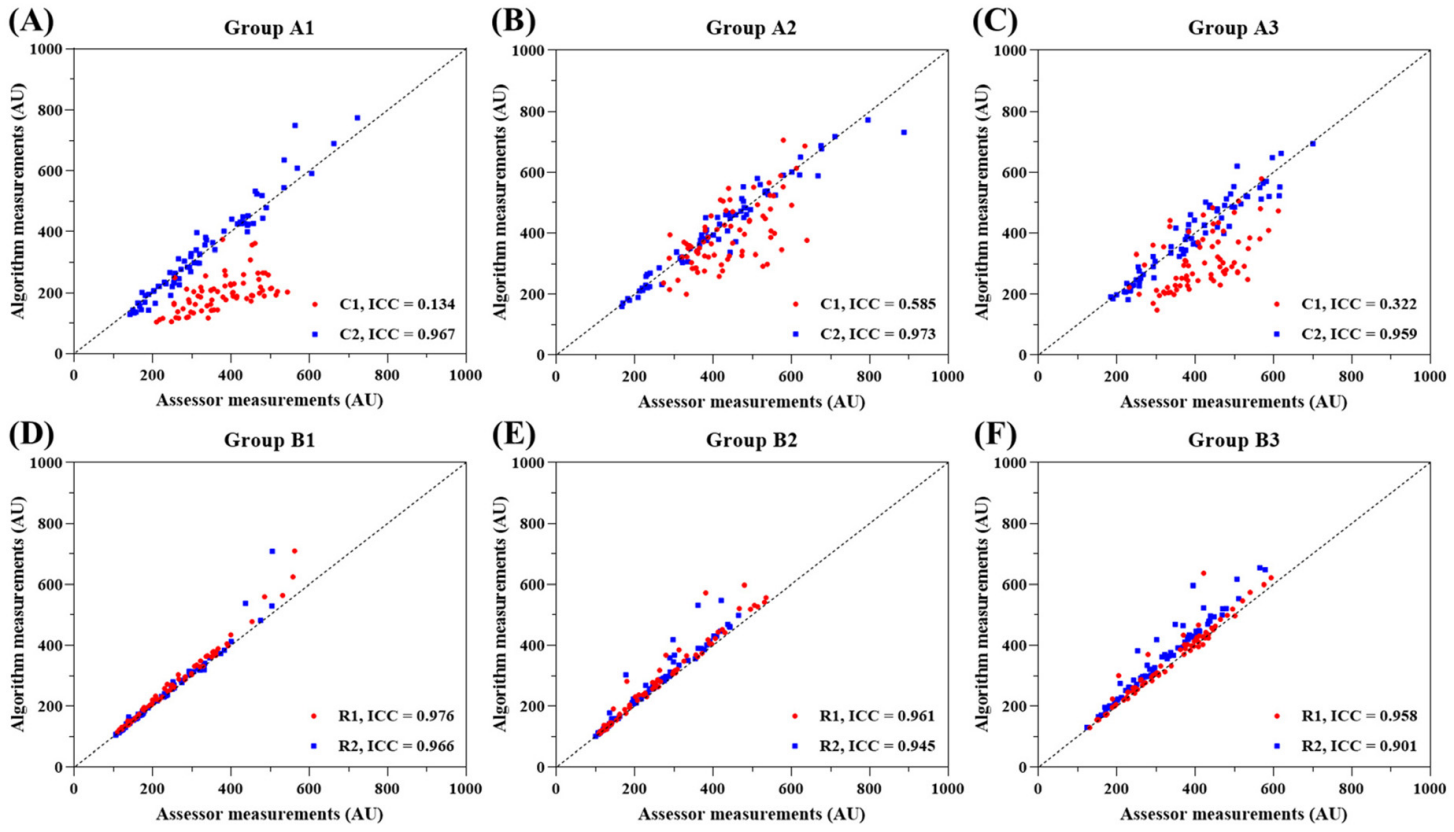
Scatterplot of the measured values between the assessor and the tracking algorithm. The scatterplot distribution showed a positive correlation between the manual and automatic tracking algorithms. (a)–(c) Results of the back of the hand. The range of the blood flow signal intensity measured by the manual tracking algorithm was 142–887 (AU), and the range of the blood flow signal intensity measured by the automatic tracking algorithm was 104–774 (AU). The ICCs of ROI C1 (ICC = 0.134–0.585) and ROI C2 (ICC = 0.959–0.973) were calculated. The ICC of ROI C1 was low, while the ICC of ROI C2 was high. (d)–(f) Results of the forearm. In the arm image, the range of the blood flow signal measured by the manual tracking algorithm was 100–594 (AU), and the range of the blood flow signal measured by the automatic tracking algorithm was 100–709 (AU). The blood flow signal ranges of ROI R1 and ROI R2 were comparable. The ICCs of ROI R1 (ICC = 0.958–0.976) and ROI R2 (ICC = 0.901–0.966) were calculated. The ICC values of the two ROIs in the three experiments were very good, with both being greater than 0.9.

Figure 1 displays the scatterplots and ICC results based on the blood flow measurements obtained during the manual and automatic tracking experiments for the three sets of images of the back of the hand [Figs. 1(a)–1(c)] and the forearm [Figs. 1(d)–1(f)]. The scatterplots show that the manual tracking and automatic tracking results were positively correlated. In the images of the back of the hand, the range of blood flow signals measured through manual tracking was 142–887 (AU), while the range measured through automatic tracking was 104–774 (AU). In the images of the forearm, the range of blood flow signals measured through manual tracking was 100–594 (AU), and that measured through automatic tracking was 100–709 (AU). Notably, in the images of the back of the hand, the performance was significantly lower for ROI C1 (ICC = 0.134–0.585) than for ROI C2 (ICC = 0.959–0.973). In the images of the forearm, ROI R1 (ICC = 0.958–0.976) and ROI R2 (ICC = 0.901–0.966) both exhibited good performance.

### B. Performance of automatic tracking technology in assessing chronic and acute wounds

**FIG. 2.**
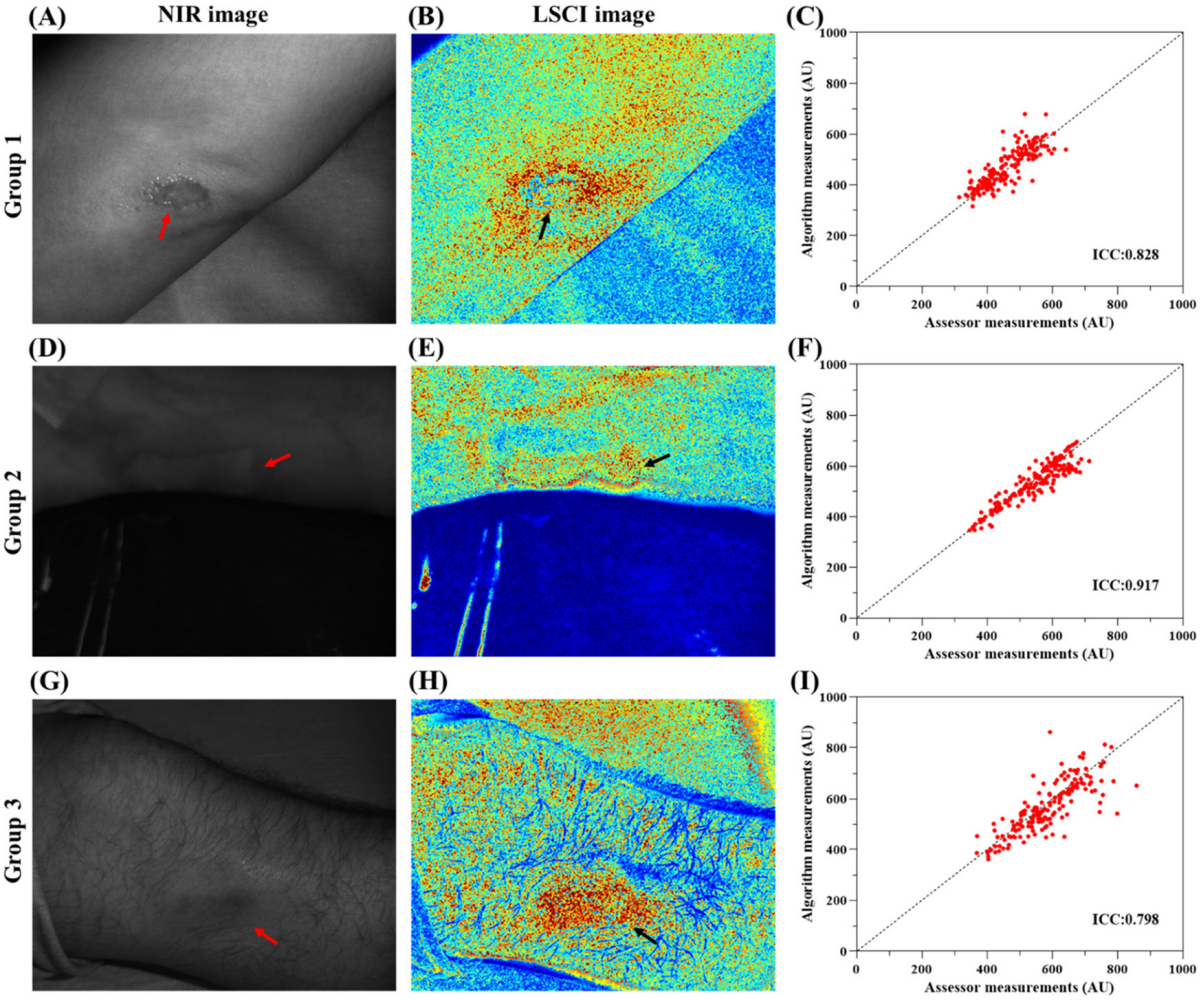
Acute wound blood perfusion measurement and tracking analysis for three different patients. (a), (d), and (g) Raw speckle images of the wounds. (b), (e), and (h) Speckle contrast images of the wounds. (c), (f), and (i) Scatterplots of the measured values based on the wound images. In the speckle contrast images, there was a significant difference between the blood perfusion in the wound area and that in the surrounding healthy tissue. For acute wounds, the range of the blood flow signal measured by the manual tracking algorithm was 315–857 (AU), and the range of the blood flow signal measured by the automatic tracking algorithm was 315–862 (AU). The ICCs ranged from 0.798–0.917. The ICCs of the three groups of wound data were greater than 0.75.

**FIG. 3.**
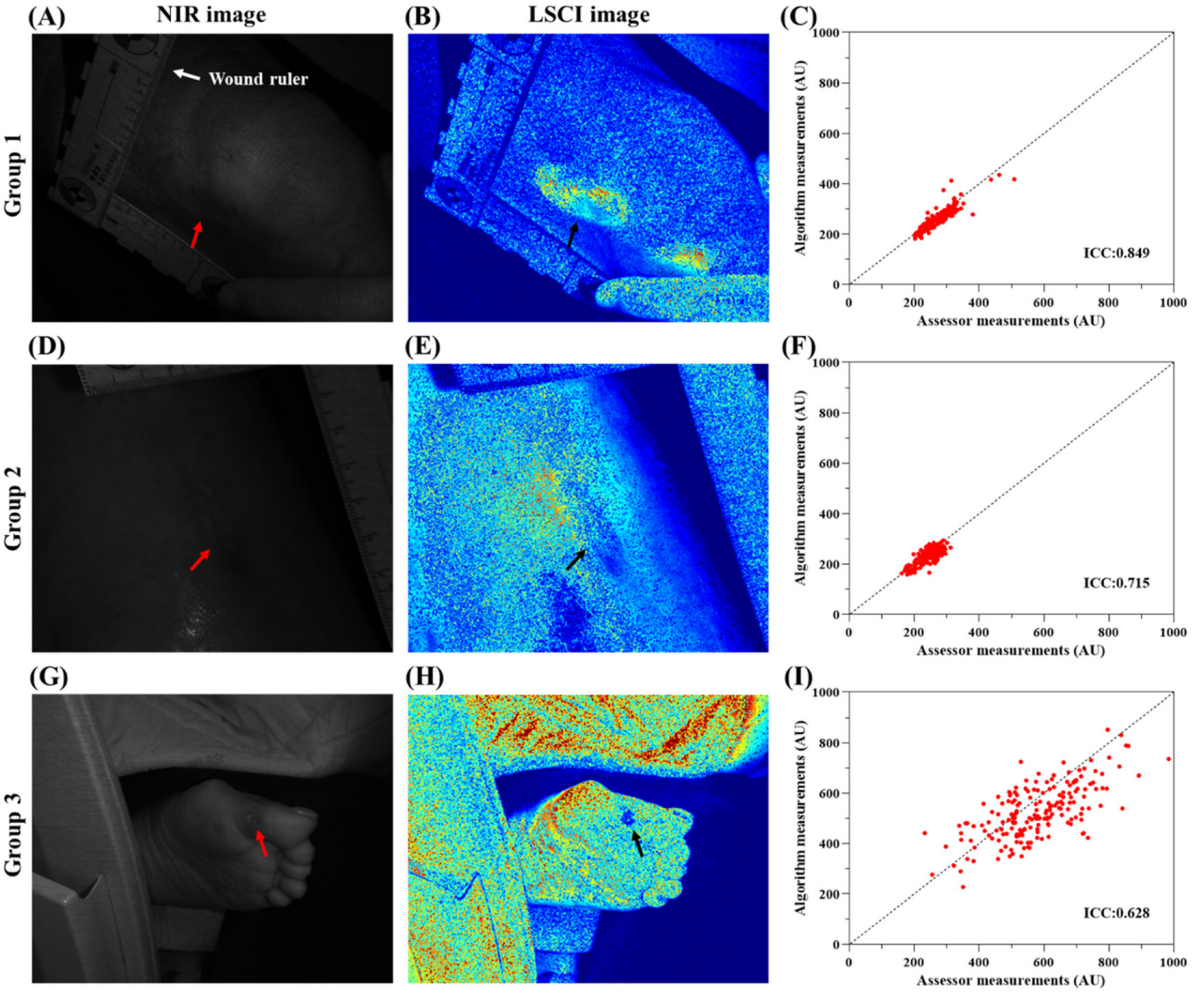
Chronic wound blood perfusion measurement and tracking analysis for three different patients. (a), (d), and (g) Raw speckle images of the wounds. (b), (e), and (h) Speckle contrast images of the wounds. (c), (f), and (i) Scatterplots of the measured values based on the wound images. For chronic wounds, the range of the blood flow signal measured by the manual tracking algorithm was 161–985 (AU), and the range of the blood flow signal measured by the automatic tracking algorithm was 158–913 (AU). The ICCs ranged from 0.628–0.849. The ICCs of the three groups of wound data were greater than 0.5.

Blood perfusion in patients with acute and chronic wounds was measured and tracked. Figures 2 and 3 present the analysis results for acute and chronic wounds in different body regions. The figures show NIR images, speckle contrast images, scatterplots of wound tracking, and ICC calculations. In the speckle contrast images, noticeable differences in blood perfusion could be observed between the wound area and the surrounding healthy tissue. For acute wounds, the range of blood flow signals measured through manual tracking was 315–857 (AU), and that measured through automatic tracking was 315–862 (AU). For chronic wounds, the range of blood flow signals measured through manual tracking was 161–985 (AU), and that measured through automatic tracking was 158–913 (AU). The ICC analysis indicated higher consistency in acute wounds (ICC = 0.798–0.917) than in chronic wounds (ICC = 0.628–0.849). The ICC values for acute wounds were all above 0.75, while the ICC values for chronic wounds ranged from 0.5 to 0.9.

## III. DISCUSSION

### A. Comparison of the advantages and disadvantages of the CSRT

Object tracking consists of two components: object detection and object tracking. Their functions are to locate objects in an image and determine whether the objects detected in consecutive frames represent the same object. Early object detection methods relied on computer vision techniques such as histograms of oriented gradients (HoGs). However, with the development of deep learning, the most commonly used object tracking algorithms include you only look once (YOLO), single-shot detectors (SSDs), and region-based convolutional neural networks (R-CNNs). In terms of implementation, these approaches generally perform well in simple environments. However, deep learning algorithms have significant time and computational resource requirements, and substantial image data are needed. Insufficient data are a key challenge that makes deep learning algorithms unsuitable for ROI tracking in speckle contrast images. Currently, there are multiple wound image databases that contain many wound images and related pathological data. The imaging modalities used to acquire these databases include color imaging, thermal imaging, and depth imaging. However, the physical properties of these types of images differ from those of speckle images, making it difficult to directly apply trained models to LSCI systems. Additionally, the number of clinical speckle contrast images of wounds is limited. Even when data augmentation techniques are applied to address the lack of training data, there is still a risk of overfitting, which significantly increases the difficulty of research in this field.

To address this limitation, we applied the CSRT object tracker in OpenCV. One of the advantages of this tracker is that during the object tracking process, users can manually select a specific region to determine the object to be detected, which increases the versatility of ROI selection. Additionally, OpenCV's object tracking method is robust against occlusion and movement. Thus, the tracker maintains its position within the ROI during movement, preventing situations in which the target is not detected. However, this also presents a drawback. Since the tracker's tracking area is the minimum rectangle enclosing the ROI, the tracking area includes some background. Due to this accumulation of errors, if the target moves out of the frame, the tracker may continue to track a portion of the background, resulting in the target disappearing while the ROI remains visible in the frame.

### B. Correlation analysis of the manual and automatic tracking methods

The experimental results for the automatic tracking performance are shown in Fig. 1. The ICC results in the scatterplot show that different measurement sites impact the consistency between the various tracking methods. When the ROI is located at the fingertip, the intensity of the blood flow measured by the automatic tracking method, represented by ROI C1, is mainly distributed below the diagonal line. This indicates that the blood flow measured by the automatic tracking method is generally less than that calculated by the manual tracking method. The ICC values for this ROI indicate poor performance, ranging from 0.134–0.585. Based on the analysis of the experimental data and images, we explain the subpar performance of the automatic tracking algorithm as follows.

**FIG. 4.**
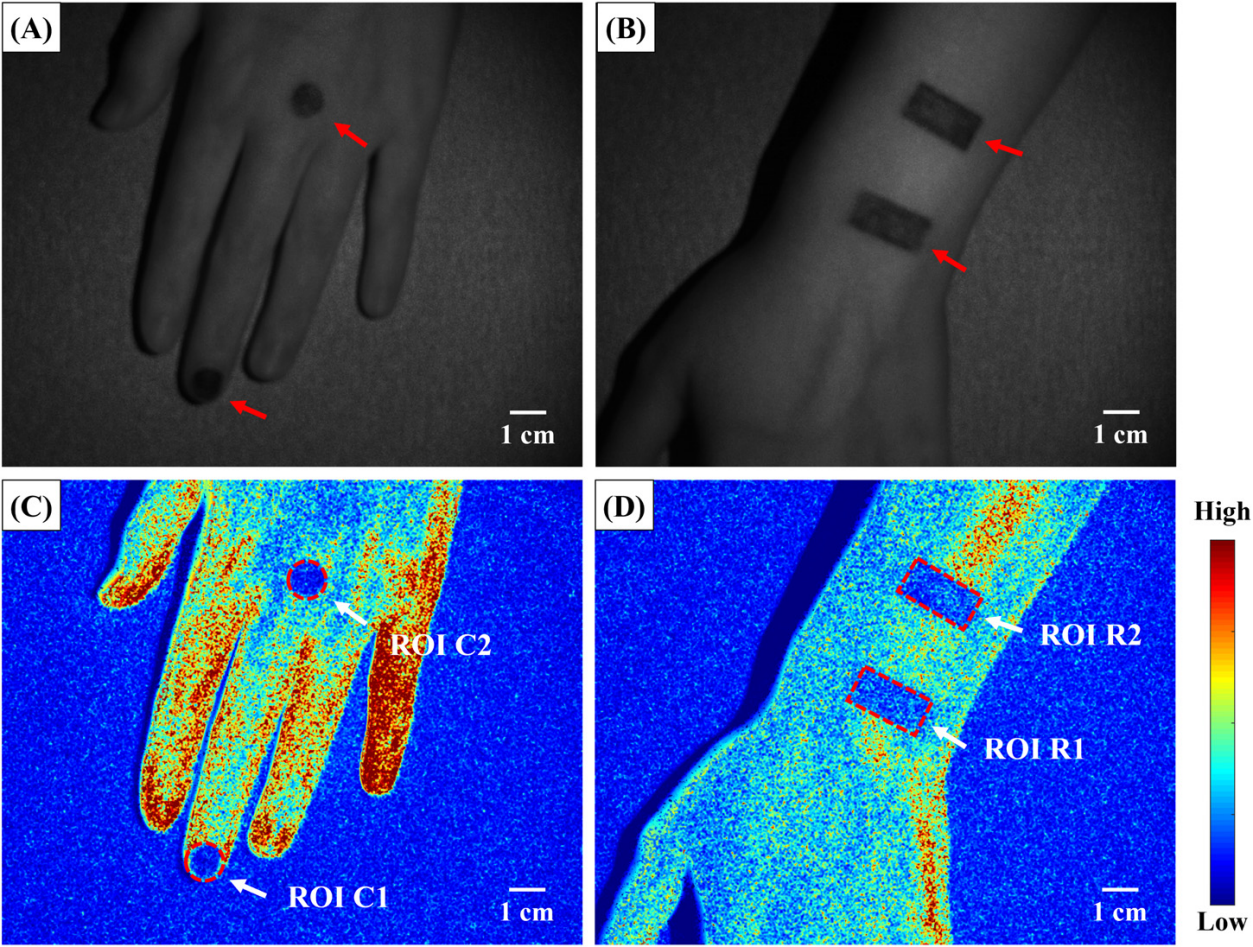
Examined skin regions and locations for AOPs applied to the skin to help replicate the disparity in blood flow between the wound area and the adjacent healthy tissue and study how the shape of AOPs and their positions influenced tracking accuracy. (a) There are two circular AOPs on the skin area located at the back of the hand. (b) On the forearm, there are two rectangular AOPs located on the skin. Additionally, there are two circular AOPs with a diameter of 1 cm on the back of the hand. One is located on the middle finger tip, and the other is located on the metacarpophalangeal joint. Two rectangular AOPs measuring 1 × 2 cm were attached near the wrist on the forearm, with a distance of approximately 2 cm between them. (c) A visual depiction of ROIs located on the back of the hand. These ROIs are labeled ROI C1, ROI C2, ROI R1, and ROI R2 based on the shape of the associated optic patterns. (d) A visual representation of an ROI on the forearm. The ROI areas are labeled ROI C1, ROI C2, ROI R1, and ROI R2 based on their shapes.

In this study, we utilized the CSRT algorithm to identify the center point of the tracking bounding box. Then, we repositioned the ROI based on the coordinates of this center point. Since the size of the CSRT bounding box changes as the target object varies, when the target object is deformed or rotated, the obtained center point of the bounding box deviates from the actual center of the target to some extent, resulting in varying degrees of ROI displacement. At this point, the size and position of the ROI may impact the automatic tracking performance. When the ROI is displaced, the calculated average intensity within an ROI with the same displacement reveals that the intensity difference is smaller for larger ROI areas than for smaller ROI areas. This is because the displaced area occupies a smaller proportion of the ROI; thus, the average intensity is closer to the correct value. In the experiment, we used AOPs to occlude blood flow. Theoretically, under the same environmental and experimental conditions, the average intensity values in the AOPs regions should be similar. During the experiment, the AOP was attached to different areas of the hand, where the surrounding skin tissue had different blood flow rates and velocity distributions. Therefore, when the bounding box was displaced, the background values with different intensities affected the calculation of the average intensity in the ROI to varying degrees. A comparison of Figs. 4(c) and 4(d) indicates that the circular ROI, with an area of 0.785 cm^2^, was smaller than the rectangular ROI, which covered an area of 2 cm^2^. Additionally, ROI C1 included not only the skin around the fingertip but also a black pad as part of the background. Therefore, the average intensity measured by the automatic tracking method was reduced due to these factors.

The speed of object movement also affects the tracking performance. If the object moves too quickly, the positional differences between adjacent frames can be significant, increasing the difficulty of accurately estimating the object's position and introducing errors. The CSRT predicts the position of the target in the current frame based on the target's position in the previous frame. This algorithm can accumulate calculation errors, leading to deviations from the actual target position. Therefore, when the object moves at a high speed, the tracking algorithm may lag, causing the bounding box of the tracked target to gradually deviate from that of the actual target. Furthermore, high-speed object movement can lead to image blur and distortion, further increasing the difficulty of tracking. In the experiments in this study, ROI C1 was displaced at a relatively fast speed, and its ICC values were significantly lower than those of the other ROIs.

### C. Speckle contrast images of acute and chronic wounds

Figures 2 and 3 show the original images, speckle contrast images, and ICC wound tracking results for acute and chronic wounds. The blood perfusion in a wound area differs significantly from that in the surrounding tissues, and the type of wound, wound severity, and healing stage all affect the level of blood perfusion. According to Fig. 2(b), 1(e), and 1(h), the blood perfusion in the wound area was lower than that in the surrounding tissues, indicating significant scab formation or open epidermal damage in the wounds. On the other hand, in Figs. 2(e), 2(h), and 1(b), the blood perfusion in the wound area was higher than that in the surrounding tissues, indicating the occurrence of healing processes. These results are consistent with the stage of blood vessel proliferation during wound healing.

### D. Characteristics of speckle contrast images of wounds for automatic tracking performance

The ICC results for acute and chronic wounds ranged from 0.628–0.917, indicating moderate to excellent performance. When comparing the performance with acute and chronic wound images, we can see that the ICC values were not significantly correlated with the wound type but were related to the clarity of the wound contour in the speckle contrast images. When the contour of the wound was more distinct, the region had higher image contrast with the surrounding tissues, resulting in lower ICC values. In the speckle contrast images, the intensity represents the variation in tissue blood flow velocity, with strong image contrast indicating a significant difference in blood flow velocity. Based on previous experimental experience, although the tracking method can be used to reposition the ROI in each frame, slight deviations may occur. In speckle contrast images with strong contrast, these deviations can lead to larger intensity differences when calculating the average intensity in the ROI, leading to lower ICC values.

### E. Future work

The tracking algorithm developed in this study can be improved. In the ROI localization and tracking process, only the target's fixed-plane translation is considered. As a result, when the target is rotated or scaled, the ROI cannot be adaptively rotated or scaled, which leads to a misalignment of the ROI position during tracking or a misalignment between the rectangular ROI and the object. To address this issue, future work should incorporate target angle and scale information into the algorithm, which may improve the automatic tracking performance. Additionally, due to the computational requirements and program execution speed of the CSR-DCF algorithm, the frame rate of the image output is limited. Therefore, the program's computations should be optimized. This can be achieved by implementing multithreading and parallel processing techniques to enhance computational efficiency and reduce program execution time.

**FIG. 5.**
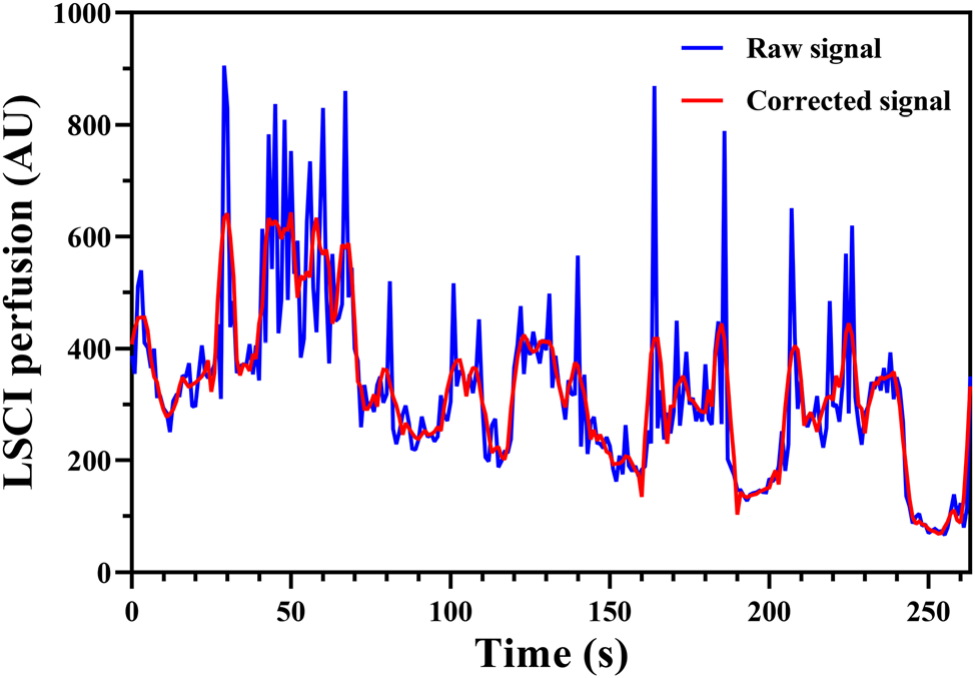
Signal calibration diagram. Based on relevant literature and previous experience, we performed postprocessing calibration based on the collected signals to ensure that the signal variations were consistent with real blood flow velocities. The blue curve represents the raw signal without any processing. This signal contains irregular signals with large amplitudes, which distort the main signal. Filtering the signal allows us to detect and eliminate motion artifacts. The filtered signal is shown by the red curve in the figure, and the instantaneous signal changes are eliminated. This approach effectively reduces the interference of motion artifacts on the blood flow signal, leading to more accurate results.

Moreover, we did not consider that LSCI systems are sensitive to image blurring caused by external motion, and the large amount of noise caused by motion artifacts may lead to differences between the LSCI blood flow estimation results and real blood flow variations. To address this issue, we can refer to the related literature^21,22^ and perform postprocessing correction based on the collected signals to ensure that the signals are accurate and consistent with the real blood flow velocity results. The changes in the values after compared with before correction are shown in Fig. 5. The blue curve shows the original signal, which contains motion artifacts. This noise can be removed by using appropriate filters, such as adaptive, average, high-pass, and low-pass filters. The red curve in the figure represents the signal after processing with the average filter. The filtered waveform is smoother than the original waveform, and no obvious amplitude signals can be observed.

Additionally, we noted that the system could not exclude background pixels. Thus, the ROI should be carefully selected to ensure that background areas outside the target are not included, which may affect blood flow analysis. Based on the characteristics of speckle contrast images, we know that stable and highly absorptive backgrounds do not cause blurring in speckle patterns. Therefore, there is a significant difference in the K values between the background and the biological tissue. Exploiting this characteristic, we placed a black light-absorbing cloth on the plane to use as the background, creating a strong contrast in the speckle patterns between the foreground and the background. By applying thresholding techniques, we successfully segmented the foreground and background. However, this method imposes limitations on the usage of LSCI, such as potential interference from clothing or sensors attached to the body during operation, which can affect the segmentation results. Therefore, we propose an alternative improved method that uses edge detection to detect the shape contours of the hands and feet and calculates the speckle contrast within the contours to obtain speckle contrast images of these regions.

## IV. CONCLUSION

Wound microcirculation monitoring is crucial for assessing wound recovery and treatment progress. Traditional methods often require manual tracking of each frame by healthcare professionals, which is time-consuming, labor-intensive, and prone to subjective errors. This study proposes an automatic tracking method based on the CSRT algorithm applied to laser speckle contrast images of wounds. The developed LSCI system in this study utilizes optical components, such as an NIR laser, diffuser, and camera for imaging, and it has been designed to be lightweight and portable. To ensure the clinical applicability of the LSCI system, it underwent testing and adjustments using the CSRT algorithm after imaging. This enables successful measurements during clinical observations, especially for patients who have difficulty maintaining a stable posture, effectively enhancing the clinical utility of the LSCI system. The experimental and analysis results show that the tracking algorithm can accurately and objectively track wound microcirculation, improving the accuracy of blood flow assessment. In the study, manual and automatic analyses were performed based on each dataset, and the ICC was calculated to evaluate the consistency between the two methods. In the wound simulation experiment, we investigated the influence of wound shape and location on the algorithm's tracking ability. The results (Fig. 1) demonstrate that wound shape does not affect the tracking ability of the developed CSRT, but the wound location in the body has a significant impact on the ICC. We applied this algorithm to real images of acute and chronic wounds, and the results (Figs. 2 and 3) show that the developed tracking algorithm is more reliable (ICC = 0.628–0.917) than manual assessment methods. In the future, we hope that this approach can be effectively applied in various LSCI-related studies and clinical trials to potentially enhance the accuracy of LSCI measurements and assessments.

## V. METHODS

### A. Basis of LSCI for blood flow visualization

Laser speckle is a random phenomenon that occurs due to the interference of scattered laser light. When laser light illuminates biological tissue surfaces, cells absorb and scatter the light, resulting in a speckle pattern with random variations in intensity and phase. Due to the optical properties of biological tissue, the motion and morphological changes of cells and tissues can cause alterations in the speckle pattern. By measuring the variations in brightness and darkness within the speckle pattern, information about tissue motion and morphological changes can be extracted.

In tissue, when red blood cells move slowly, the variations in brightness within the speckle pattern are smaller, and the pattern becomes more blurred. Conversely, when the red blood cells move faster, there are larger fluctuations in brightness within the speckle pattern, leading to a sharper pattern. Based on the formula proposed by Goodman,^23^ the blurring of the speckle pattern can be quantified as speckle contrast. The speckle contrast, denoted as K, is defined as the ratio of the standard deviation σ of the speckle intensity to the average speckle intensity $\left\langle I\right\rangle$ and can be calculated as shown in the following equation:

$$K=\frac{\sigma }{\left\langle I\right\rangle }=\frac{\sqrt{\left\langle I^{2}\right\rangle -{\left\langle I\right\rangle }^{2}}}{\left\langle I\right\rangle }.$$ (1)

Fercher and Briers were the first to apply laser speckle for blood flow measurements.^24^ They utilized the findings of Goodman and successfully obtained vascular images of the retina using laser speckle flowmetry (LSF), which allowed for the quantification of blood flow velocity to some extent.

LSCI is a noninvasive optical technique for imaging blood flow. LSCI yields high spatial and temporal resolution measurements of dynamic blood flow by determining the correlation between the speckle contrast and the velocity of the scattered particles. LSCI was initially proposed by Briers.^25^ LSCI systems record laser speckle images using CCD cameras, and the resulting images can be used to analyze the influence of factors such as blood flow velocity and red blood cell density on hemodynamics. The relationship between speckle contrast and blood flow velocity can be expressed as follows:

$$\frac{1}{K^{2}}\propto v.$$ (2)

This relationship indicates that speckle contrast and blood flow velocity are correlated. By measuring the magnitude of the speckle contrast, the changes in blood flow velocity can be indirectly estimated. This allows for noninvasive, high-resolution, and large-area blood flow imaging. Thus, LSCI is a valuable tool for assessing blood flow dynamics in various biomedical and clinical applications.

### B. Portable system for LSCI and data presentation

**FIG. 6.**
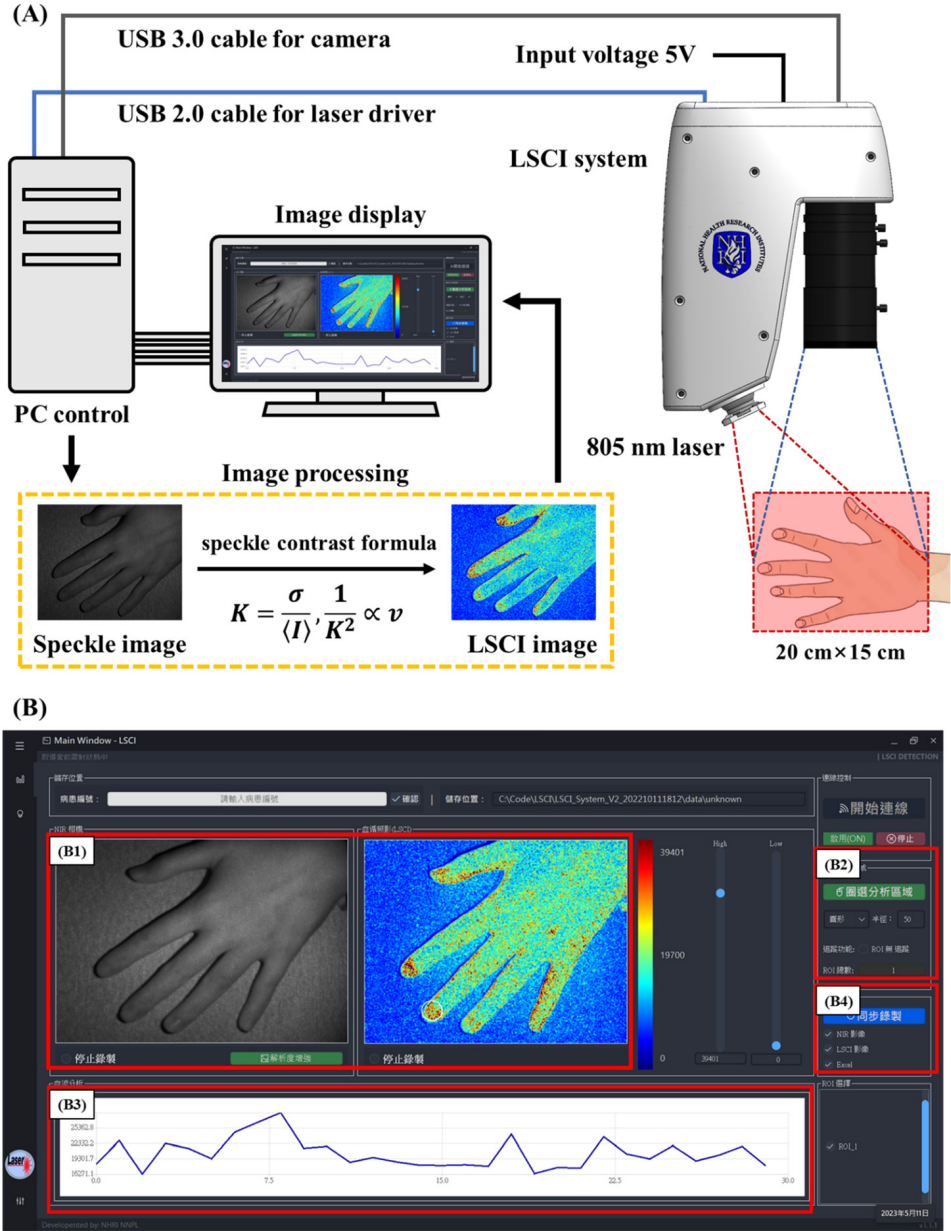
Implementation of a portable LSCI device. (a) Schematic diagram of the LSCI experiments. In this study, we designed a portable device to acquire image data. The system utilized an 805-nm NIR laser with a power of 600 mW to illuminate the skin. The CMOS camera (acA1300-200 *μ*m, Basler, Germany) acquired images with a resolution of 1280 × 1024 pixels in an exposure time of 5 ms. During image data acquisition, the distance between the lens and the skin was maintained at 30 cm, corresponding to an imaging area of 20 × 15 cm. Under 805-nm laser illumination, monochromatic speckle images were obtained. We applied LASCA theory to process the speckle images and obtained color-coded speckle contrast images. (b) The graphical user interface (GUI) of the system. In this study, we provided users with a concise and intuitive GUI to facilitate the operation of the LSCI system and reduce complexity. The interface displays both the current raw image and the speckle contrast image and provides circular and polygonal ROI selection functions for users to analyze blood flow intensity. It also includes features for image and data storage.

Figure 6 illustrates the implementation of the LSCI system used in this study. A portable device was designed to acquire speckle images, as shown in Fig. 6(a). The system employed a laser driver (SF8075-NM, Maiman Electronics, Russia) to control a near-infrared (NIR) laser (ML620G40, Thorlabs, USA) with a wavelength of 805 nm, which was directed onto the skin surface with a power of 600 mW. To achieve uniform illumination, a circular pattern diffuser (ED1-C50, Thorlabs, USA) was placed at the laser output, causing light to diverge at an angle of 50 degrees, thus ensuring a circular and uniform light distribution. The device utilized a CMOS monochrome camera (acA1300-200 *μ*m, Basler, Germany) and a macro zoom lens (MLH-10X, Computer, Japan) to record images with a resolution of 1280 × 1024 pixels. The exposure time for the images was set to 5 ms. During the experiments, the distance between the lens and the skin was maintained at 30 cm, which allowed for an imaging area of 20 × 15 cm. Under these conditions, the energy was uniformly dispersed through a diffuser, resulting in a power range of 238.3–525.1 *μ*W reaching the target, with 96.7% of the field of view (FoV) falling within the full width at half maximum (FWHM). Illumination with the NIR laser was used to obtain monochromatic speckle images. The system also performed image processing, utilizing Eqs. (1) and (2) to analyze the images collected by the LSCI system. Subsequently, a linear color map was applied to visualize the images. In the color map, high blood flow (low speckle contrast) is displayed in red, while low blood flow (high speckle contrast) is displayed in blue.

To improve the convenience for clinical operators and reduce operational complexity, we integrated the image processing and system control operations into operating software based on the Windows system. The software was developed using Python. Figure 6(b) illustrates the software interface, which includes several functional windows. The first window presents the current raw image and speckle contrast image (B1). The speckle contrast image can be adjusted using the slider on the right to modify the color distribution threshold. To configure the image analysis area (B2), users can choose between a circular ROI or a polygonal ROI and select whether to use the tracking function. The blood flow analysis results within the ROI area (B3) are displayed as a line graph. To assess the image and data recording functionality (B4), the software records both the raw image and speckle contrast image, and the blood flow analysis results can be exported in Excel format.

Speckle contrast images can be obtained based on laser speckle contrast analysis (LASCA) theory. There are two main methods for performing calculations based on speckle contrast images: spatial estimation and temporal estimation.^26^ In this study, to achieve sufficient temporal resolution, based on the computational speed of the system, the spatial estimation method was chosen to calculate the speckle contrast for each image frame. However, this method involves independent calculations based on fixed-size regions surrounding individual pixels, resulting in reduced spatial resolution. As a result, this approach may not effectively detect subtle spatial variations.

**FIG. 7.**
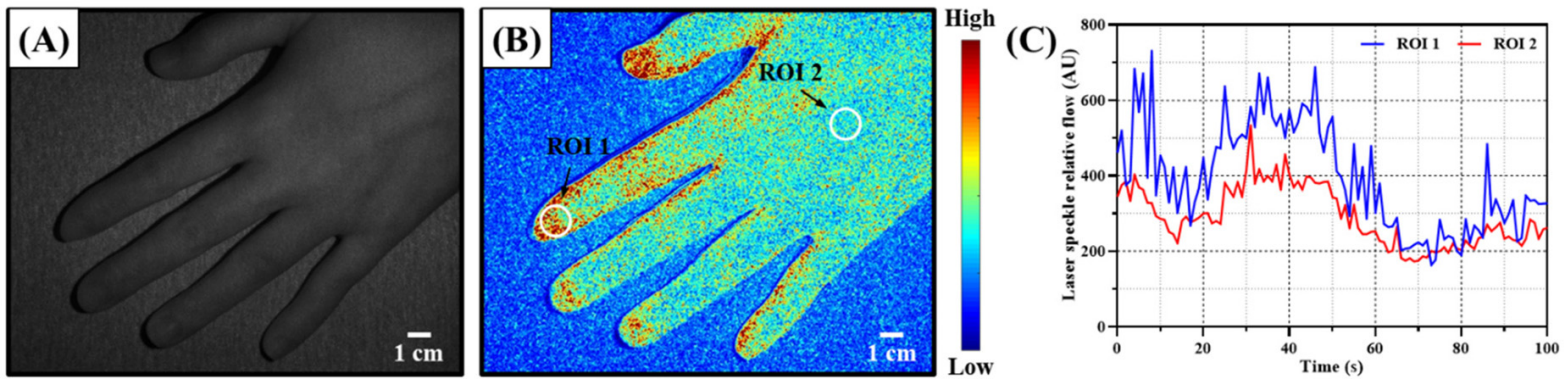
Image processing and data presentation for the hand region. (a) Raw image. The original speckle pattern was obtained by illuminating the hand with an 805-nm laser. (b) Speckle contrast image. By calculating the speckle contrast, the blood flow distribution information in the hand can be visualized, and ROIs can be selected for perfusion analysis. (c) The perfusion signals in the selected ROIs as indicated in (b). The perfusion signals in the chosen ROIs changeover time.

Figure 7 illustrates the images and data obtained by the implemented system. Figure 7(a) displays the original grayscale intensity image captured by the camera. After uniform illumination with 805-nm laser light, a random speckle interference pattern is formed. Through speckle contrast image processing, a blood flow speckle contrast map of the hand can be generated, as shown in Fig. 7(b). In the image, red represents faster blood flow, while blue represents slower blood flow. The fingertip exhibits higher blood flow volume and density. By selecting the ROI in the speckle contrast image, blood flow signals can be acquired. The software plots the signal variations within the selected region as a line graph for analysis, as shown in Fig. 7(c). The graph shows that different ROIs exhibit distinct variations in blood perfusion.

### C. Development and implementation of ROI tracking in LSCI systems

Users often apply LSCI systems to measure and analyze blood flow in specific regions. However, various factors, such as muscle contractions and user-induced movements, can displace the measurement targets in the blood flow images. These displacements can affect the image quality and blood flow analysis accuracy, thereby impacting the clinical applicability of LSCI systems. Therefore, to address this issue, an automatic ROI tracking feature is needed. This feature can automatically detect and correct displaced ROIs, ensuring good image quality and high accuracy in blood flow analysis and ultimately enhancing the clinical utility of LSCI systems.

**FIG. 8.**
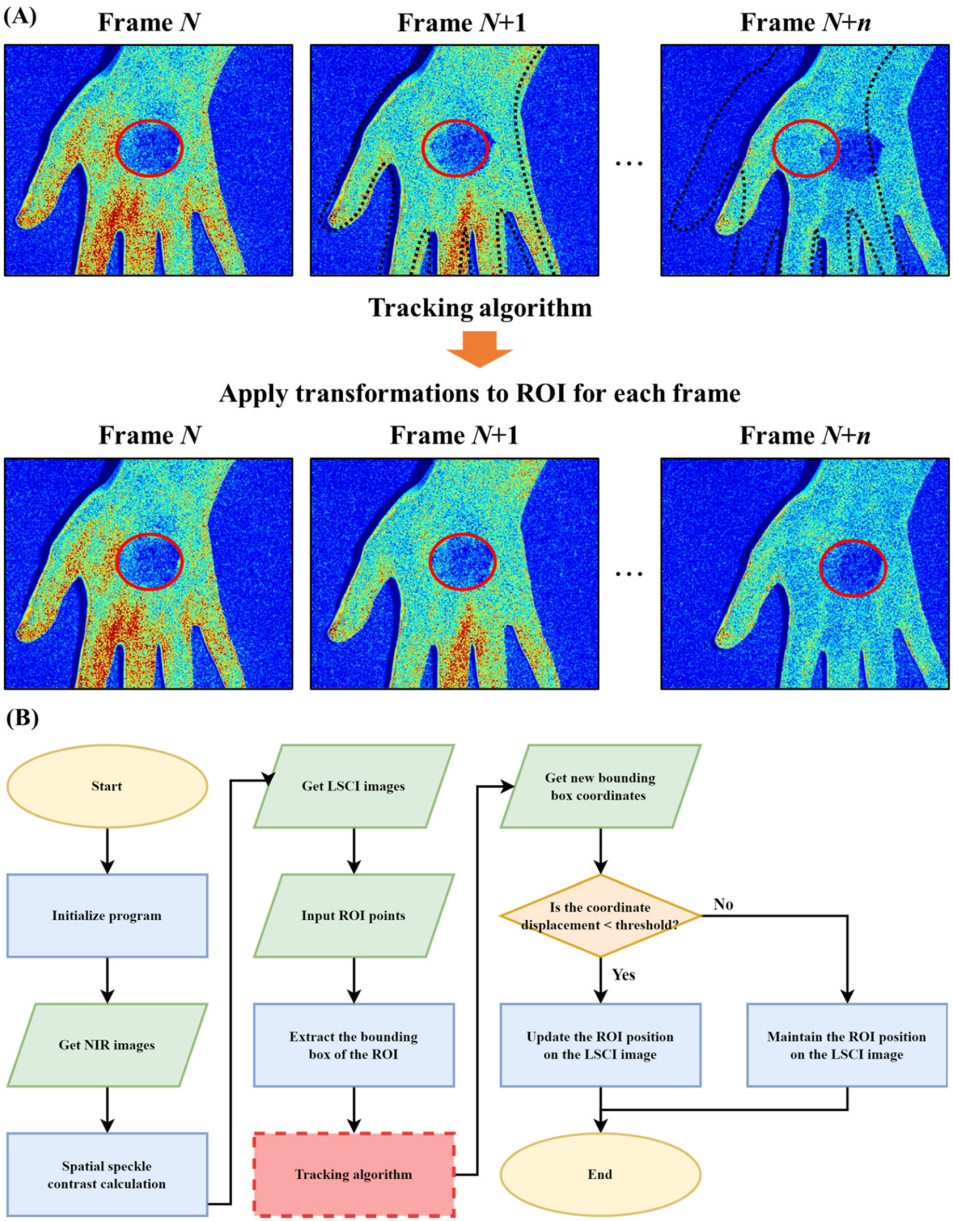
A step-by-step diagram that illustrates the algorithm for ROI tracking. (a) When using the LSCI system, the ROI remains fixed at its initially drawn coordinates, but certain human factors may cause the lesion location to shift. In such cases, the user must reselect the lesion location, which can be a time-consuming process. To simplify this process, the ROI tracking principle is employed. It involves obtaining the ROI's (red contour line) geometric coordinates and the internal image features from the Nth frame and using this information to predict the ROI's position in the N + 1th frame. The ROI area is then updated based on the predicted position. In this figure, the LSCI image change is depicted for the practice tracking algorithm. As the palm moves toward the right, the ROI will accurately adjust its position to the lesion. (b) We start by initiating the ROI tracking operation and importing the NIR image. Next, we calculate the scatter contrast intensity of the image and obtain the LSCI image. Then, we plot the ROI and gather the coordinates of each point in the ROI. After creating the bounding box, we use a tracking algorithm for target tracking. If the displacement of the bounding box exceeds the set threshold, we consider the object to be moving and update the ROI coordinates. Conversely, if the displacement is below the threshold, we consider the object stationary and maintain the ROI's original position.

Figure 8(a) illustrates a schematic diagram of the proposed ROI tracking method. In the operation process of the LSCI system, the user manually selects an ROI in the sampled image, typically representing a lesion area (indicated by the red contour), for analysis. Under normal circumstances, the ROI remains fixed at the initially designated coordinates. However, factors such as hand or foot tremors and body respiratory movements can change the lesion's position, while the ROI remains stationary, leading to failures in tracking spatial variations. In such cases, the user needs to reselect a suitable ROI or manually adjust the ROI position. To address this issue, object tracking techniques can be used. The image processing principle of ROI tracking involves obtaining the ROI delineated by the user in the Nth image frame. Then, the geometric coordinates and internal image feature information of the ROI, such as the color, texture, and shape, are extracted. Based on the feature information obtained from the Nth frame, the predicted position of the target ROI in the N + 1th frame can be estimated. Finally, the estimated target position is used as the new target center, and the ROI position is updated. The changes in the speckle contrast image during the implementation of the tracking algorithm are shown in the figure. When the palm moves to the right, the ROI moves to the correct position of the lesion (video available in the supplementary material).

Figure 8(b) shows a flow chart of the ROI tracking operation. At the start of the experiment, the program settings are initialized. Then, the NIR image is captured by the camera, and the speckle contrast calculation is performed based on the image to obtain the speckle contrast image. Then, the ROI is delineated on the image. A bounding box is delineated based on the circumscribed rectangle of the ROI, and the tracking algorithm is used to track the target according to the concept shown in Fig. 8(a). When the tracking is successful, new bounding box coordinates can be obtained. During the experiment, we found that even if the target object does not move, there is still a slight offset between the bounding box of the current frame and the bounding box of the previous frame, leading to irregular jitters in the ROI delineated in the image. Therefore, we define that the object has moved when the displacement between the bounding boxes of the current and previous frames is greater than a certain value, and the ROI position in the speckle contrast image is subsequently updated.

### D. CSRT algorithm

This study utilized the CSRT algorithm for ROI tracking.^27^ The CSRT is an object tracking algorithm based on the discriminative correlation filter with channel and spatial reliability (CSR-DCF) that is included in the OpenCV library.^28,29^ The DCF method extracts features from the target region in the first frame and is trained to obtain the correlation filter. For each subsequent frame, features are extracted from the predicted region in the previous frame, and correlation operations are performed with the filter to predict the target's position in the next frame. By continuously repeating these steps for subsequent target tracking and model training, real-time target tracking can be achieved.

However, the DCF method has some limitations. Because the fast Fourier transform (FFT) is used, the size of the filter and the patch must be the same, restricting the size of the target detection region. Additionally, fast target motion or deformation can lead to target fragmentation and boundary effects, which can degrade the effectiveness of the learned tracking model. The CSRT introduces spatial and channel reliability to the DCF framework. Spatial reliability involves adding a mask matrix related to the target to the filter in a spatial manner. This mask matrix suppresses boundary effects and enhances tracking performance for nonrectangular targets. The mask matrix is constructed based on color histograms of the foreground and background. Channel reliability determines the reliability of each feature channel for target tracking by weighting different feature channels. Channel reliability can help in determining the optimal channel combination for tracking.

### E. Affected part movement simulation experiment

To evaluate the ROI tracking performance of the CSRT, we designed a simulation experiment involving the movement of a lesion. We compared the manual tracking analysis results with the automatic tracking results to evaluate the differences between the two methods. In the experiment, we used adhesive opaque patches (AOPs) attached to the skin to block blood flow, simulating a wound and creating a contrast in blood flow between the wound and the surrounding healthy tissue. Figure 4 illustrates the AOPs and ROIs on the skin for this experiment. To investigate whether the shape and placement of the AOPs affected the performance of the CSRT, we selected the back of the hand and the anterior portion of the forearm as the target areas for testing, as shown in Figs. 4(a) and 4(b). Two circular AOPs with diameters of 1 cm were attached to the fingertip of the middle finger and the knuckle joint. Two rectangular AOPs measuring 1 × 2 cm spaced by approximately 2 cm were attached to the forearm near the wrist. During the experiment, the palm and forearm of the subject were placed flat on a black light-absorbing cloth. This was performed because the background may influence the tracking performance when using the CSRT; when the background and the target have similar colors, textures, or shapes, the algorithm may mistakenly track the background as the target. Therefore, the experiment was conducted with a single background to reduce the influence of background noise, improve image contrast, obtain more accurate blood flow speckle contrast images, and reduce potential image distortion and errors. Figures 4(c) and 4(d) show the selected ROIs for evaluating the tracking performance. We considered the areas with AOPs in the selection of ROIs. Based on the shape of the selected ROIs, they were named ROI C1, ROI C2, ROI R1, and ROI R2.

In this experiment, the palm and forearm of the subjects were selected as the target areas. During the experiment, the subjects were instructed to perform repetitive and stable movements within the range of the camera. Additionally, they were asked to repeatedly move their fingers, with each movement maintained for the same time interval. The duration of the experiment was 30 s. For the manual analysis method, the ROI was manually repositioned in each frame. For the automatic tracking method, all ROIs were manually positioned in the first frame, and the ROIs in the subsequent frames were automatically positioned.

### F. ROI tracking performance evaluation using ICCs

To determine the differences between the automatic and manual tracking methods, the intraclass correlation coefficient (ICC) with a two-way random model was used to measure the consistency between the two sets of data. The ICC is a statistical measure used to assess the reliability of measurement data. It is primarily employed to evaluate the consistency of measurements made by the same rater based on the same subjects or the repeatability/consistency of measurements made by different raters based on the same subjects. ICC values range from 0 to 1, with higher values indicating greater reliability and less variation among the raters and lower values indicating lower reliability and greater variation among the raters. Generally, ICC values below 0.5 indicate poor reliability, ICC values in the range of 0.5–0.75 indicate moderate reliability, ICC values in the range of 0.75–0.9 indicate good reliability, and ICC values above 0.9 indicate excellent reliability. The ICC is usually expressed according to the following equation:

$$\rho =\frac{\sigma_{B}^{2}}{\sigma_{B}^{2}+\sigma_{W}^{2}}.$$ (3)

In the equation, $\sigma_{B}^{2}$ represents the variance of interest and $\sigma_{W}^{2}$ represents the unwanted variance.

### G. Feasibility of automatic tracking technology in assessing chronic and acute wounds

This study also explored the feasibility of applying automatic tracking techniques in clinical practice by assessing acute and chronic wounds in patients. All procedures were performed in accordance with the Institutional Review Board (IRB) of Changhua Christian Hospital, Taiwan (IRB number: 211117). For inclusion in the study, individuals were enrolled after signing an informed consent form. All participants in this study were at least 20 years old, and participants were selected regardless of sex. For inclusion in the study, individuals were enrolled after signing an informed consent form and needed to have either an acute or chronic wound and require regular care by healthcare professionals for wound cleansing and dressing changes. Chronic wound cases included patients with diabetic foot ulcers, pressure ulcers, etc., while acute wound cases included nonhospitalized patients with second-degree burns (burn area <15%), abrasions, and lacerations. The exclusion criteria for this study were as follows: (i) autoimmune diseases, (ii) organ failure, (iii) radiation ulcers or cancerous wounds, and (iv) inability to cooperate with the measurements. A total of 10 patients with acute or chronic wounds participated in this study, with five patients in each category. The LSCI measurements and ROI localization for each wound site were performed by trained researchers. Each measurement took 90 s, resulting in a total of 15 LSCI recordings. The image data for manual and automatic ROI tracking were collected simultaneously. From these 15 LSCI recordings, images with significant wound displacement were selected, with three sets of images meeting the criteria for both acute and chronic wounds. Finally, the ICCs were calculated to assess the differences between the two methods.

## SUPPLEMENTARY MATERIAL

See the supplementary material of the paper contains two Supplementary Movies demonstrating the performance of using the CSRT object tracking algorithm for LSCI system.

## Data Availability

The data that support the findings of this study are available from the corresponding author upon reasonable request.
